# The Dual Role of ADAMTS1 in Cardiovascular Remodeling: Balancing Extracellular Matrix Homeostasis and Pathological States

**DOI:** 10.3390/jcdd12120467

**Published:** 2025-11-29

**Authors:** Siqin Sheng, Shunrong Zhang

**Affiliations:** 1 The Fourth Clinical School of Medicine Affiliated to Zhejiang Chinese Medical University, Hangzhou 310059, China; 15825781243@163.com; 2 Department of Geriatrics, Affiliated Hangzhou First People’s Hospital, Westlake University School of Medicine, Hangzhou 310006, China

**Keywords:** ADAMTS1, cardiovascular remodeling, extracellular matrix, dual role, proteolysis, versican

## Abstract

Extracellular matrix metalloproteinase ADAMTS1 (adhesion metalloproteinase with thrombospondin-type domain 1) is a key regulator in cardiovascular remodeling with functional paradoxes. This review synthesizes existing evidence to clarify its context-dependent dual roles across various cardiovascular diseases: on the one hand, ADAMTS1 exerts protective functions by maintaining vascular integrity and mitigating inflammatory responses; on the other hand, in conditions such as myocardial infarction and aortic aneurysms, ADAMTS1 promotes pathological progression by excessively hydrolyzing the multifunctional proteoglycan versican and other substrates, leading to tissue disruption and adverse remodeling. This functional switch in ADAMTS1 is jointly regulated by its cellular origin, temporal expression dynamics, and local microenvironment. In summary, ADAMTS1 represents a critical homeostasis node in the cardiovascular system. Therapeutic interventions targeting it should avoid broad-spectrum inhibition strategies; instead, future efforts should focus on developing precise, context-specific regulatory approaches.

## 1. Introduction

Cardiovascular diseases (CVDs) remain among the leading causes of morbidity and mortality worldwide. Beyond the traditional focus on cardiomyocytes and hemodynamic factors, the extracellular matrix (ECM) is now recognized as a dynamic key component regulating cardiac and vascular structure, function, and signaling. The ECM is not a static scaffold but a three-dimensional network undergoing continuous, tightly regulated remodeling. When the body encounters injurious stimuli such as hypertension, ischemia, or hyperlipidemia, the steady-state equilibrium of the ECM is disrupted, triggering pathological ECM remodeling. This process is a core feature of various cardiovascular diseases, including heart failure, aortic aneurysms, and atherosclerosis [1,2,3]. ECM remodeling involves alterations in cellular composition and the abnormal synthesis, deposition, and degradation of ECM components. It may manifest as a compensatory repair response or evolve into a detrimental process driving disease progression: pathological changes such as myocardial stiffness in heart failure and fibrotic cap instability in atherosclerosis are directly linked to pathological ECM remodeling [4,5].

The complex process of ECM remodeling is primarily regulated by the zinc-dependent protease family, with matrix metalloproteinases (MMPs) and the ADAMTS (adhesion-dissociating metalloproteinase-like) family being the most critical [6,7]. Although MMPs have been extensively studied, ADAMTS proteases have only recently been recognized as important regulators of cardiovascular structure and function [8]. The human ADAMTS family comprises 19 secreted multidomain enzymes that act on various ECM components and bioactive molecules [8,9]. Based on substrate specificity, the ADAMTS family can be further classified, namely the aggrecanases or proteoglycanases (ADAMTS1, 4, 5, 8, 9, 15 and 20), the procollagen N-propeptidases (ADAMTS2, 3 and 14), the cartilage oligomeric matrix protein-cleaving enzymes (ADAMTS7 and 12), the von Willebrand Factor proteinase (ADAMTS13), and a group of orphan enzymes (ADAMTS6, 10, 16, 17, 18 and 19) [9]. Members such as ADAMTS1, ADAMTS4, and ADAMTS5 exhibit potent proteoglycanase or aggrecanase activity, capable of cleaving key arterial ECM proteoglycans (e.g., the multifunctional proteoglycan versican and aggrecan). Their canonical vascular substrate is versican, a critical determinant of vascular structure and stability. Notably, their activity extends to the cartilage-derived proteoglycan aggrecan, which, beyond its classic role in load-bearing, is increasingly recognized as a functional component in cardiovascular development and a range of pathological conditions, including aortic aneurysms and atherosclerosis [9,10,11]. These cleavage processes play central roles in developmental morphogenesis, inflammatory responses, and vascular biology. Furthermore, genetic or functional abnormalities in specific ADAMTS members have been directly linked to cardiovascular pathologic changes [7,8].

**Figure 1:**
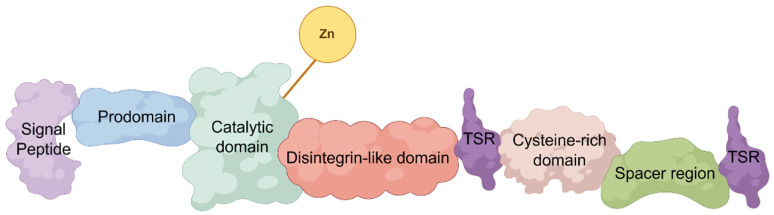
Domain organization of ADAMTS1. ADAMTS1 is a modular multi-domain protein. The structure begins with an N-terminal signal peptide (SP) for secretion, followed by a prodomain that maintains the enzyme in an inactive state. The catalytic domain, which houses the zinc-binding motif, is responsible for substrate proteolysis. Downstream domains, including the disintegrin-like module, thrombospondin type 1 motifs (TSR), a cysteine-rich domain, and a spacer region, are critical for substrate recognition, binding to extracellular matrix components, and interaction with inhibitors such as TIMP-3. The specific arrangement of these domains underpins the diverse functional capabilities and precise regulation of ADAMTS1.

Among the ADAMTS family members, ADAMTS1 holds a unique position. It was initially identified as a gene rapidly and transiently induced in the early stages of acute myocardial infarction, suggesting its role as an “immediate early response gene” to ischemic injury [12]. Functionally, ADAMTS1 is a complex protease whose activity is determined by its multi-domain architecture (Figure 1). for example, the highly variable loop within its spacer domain acts as an “exocatalytic site,” participating in substrate recognition and determining substrate specificity [10]. ADAMTS1 cleaves versican, the primary structural proteoglycan in arteries, though its enzymatic efficiency is lower than that of ADAMTS5, and its activity is tightly regulated [10]. Beyond proteolytic functions, ADAMTS1 participates in non-catalytic interactions (e.g., binding to the matrix–cell protein TFPI-2) and has been demonstrated to regulate redox signaling pathways (particularly the nitric oxide NOS2 pathway in aortic diseases) [13,14]. However, a systematic review of existing literature reveals “contradictory” and “context-dependent” roles for ADAMTS1: for instance, *Adamts1* haploinsufficient mice spontaneously develop thoracic aortic aneurysms and dissections (TAAD), indicating its critical importance for vascular homeostasis [14]; yet, in other contexts, ADAMTS1 upregulation correlates with pathological tissue remodeling, driving disease progression by promoting ECM degradation [6,8]. This duality suggests ADAMTS1 functions are not inherently “beneficial” or “harmful”, but rather determined by complex, not fully elucidated regulatory interactions. The current knowledge gap lies in systematically integrating evidence to explain why its protective function shifts to a pathological role, and which specific molecular and cellular factors govern this critical transition.

This review aims to consolidate existing evidence on the multifaceted role of ADAMTS1 in cardiovascular remodeling, focusing on its dual functions across various cardiovascular diseases such as atherosclerosis, aortic aneurysms, and heart failure. It further explores the mechanisms underlying its functional context dependence, including regulatory networks associated with transcriptional control, microenvironmental signaling, and substrate specificity. Finally, it discusses the translational potential of ADAMTS1 as a biomarker and therapeutic target, proposing future research directions to achieve clinical benefits by modulating its biological functions.

## 2. The Protective Face of ADAMTS1

Evidence indicates that ADAMTS1 functions not merely as a “destructive” protease, but rather as a key regulatory factor in maintaining vascular homeostasis. By coordinating the body’s adaptive responses to physiological and pathological stimuli, it exerts significant protective effects in vascular remodeling and the preservation of vascular integrity.

The homeostatic function of ADAMTS1 is reflected in its induction by anti-atherosclerotic hemodynamic signals. Relevant literature [15] indicated that endothelial cells exposed to extremely high wall shear stress, a known stimulus for arterial dilatory remodeling, specifically upregulate ADAMTS1 expression. This blood flow-induced ADAMTS1 expression pattern constitutes a core component of the “antithrombotic-anti-inflammatory” transcriptional program, synergistically driving the protective transformation of the endothelial phenotype. More importantly, the in vivo relevance of this function has been confirmed in a rabbit model of increased blood flow: researchers detected significantly elevated ADAMTS1 protein levels in arteries undergoing remodeling [15]. These data indicate that ADAMTS1 is a key responsive molecule in endothelial cells to high-flow stimulation, potentially maintaining vascular lumen patency by participating in compensatory outward remodeling.

Beyond its role in hemodynamic regulation, ADAMTS1 appears to function as a “gatekeeper” for vascular inflammation, with its expression deficiency associated with severe vascular events. A pivotal human study on acute Stanford type B aortic dissection revealed significantly reduced ADAMTS1 mRNA levels within circulating CD14+ monocytes in patients compared to healthy controls [16]. This finding suggests that baseline ADAMTS1 expression in specific immune cell populations may be critical for maintaining aortic wall integrity, while its absence may promote the formation of a “dissection-prone microenvironment.” This conclusion aligns with current understanding of atherosclerosis, where the plaque microenvironment is a key determinant of disease progression and stability [4]. Within this microenvironment, ADAMTS1 interacts with critical signaling pathways, such as the vascular endothelial growth factor A (VEGFA) pathway, to exert fine-tuned regulatory effects on processes like angiogenesis. Computational analysis indicates that signaling mediated by the VEGFA-ADAMTS1 complex is indispensable for organized angiogenesis [17]. This suggests that ADAMTS1 may function not only to limit the quantity of new blood vessels but also to regulate their quality, thereby preventing the formation of leaky, immature vessels that exacerbate plaque instability.

In summary, the protective role of ADAMTS1 manifests primarily through two key characteristics: First, as a molecule responding to shear stress in endothelial cells, it promotes adaptive vascular remodeling [15]. Second, as a stability regulator, its expression deficiency in monocytes correlates with acute aortic syndrome [16]. The dual function of ADAMTS1 is integrated into the complex equilibrium of the plaque microenvironment [4], where it may ensure orderly tissue repair processes through interactions with key signaling axes such as VEGFA [17]. These insights position ADAMTS1 as a critical component of the vascular system’s intrinsic defense mechanism, which helps vessels withstand damage from both mechanical stresses, such as high shear stress, and inflammatory stress.

## 3. The Pathological Face of ADAMTS1

### 3.1. In Atherosclerosis

During the progression of atherosclerosis, ADAMTS1 primarily exerts pathological effects, specifically by promoting plaque vulnerability and driving adverse vascular remodeling. Strong clinical evidence from human studies corroborated this function: compared to patients with stable angina, ADAMTS1 expression is significantly elevated in unstable coronary plaques from acute myocardial infarction patients, with its localization colocalizing with macrophages and the cleavage site of the multifunctional proteoglycan versican [18]. Data from carotid endarterectomy specimens further strengthen the association between ADAMTS1 and plaque instability, with studies demonstrating that mRNA expression of ADAMTS1 is upregulated in unstable lesions, while expression of its inhibitor, tissue inhibitor of metalloproteinase 1 (TIMP-1), is correspondingly reduced [19].

The mechanism by which ADAMTS1 produces these harmful effects manifests primarily in two ways: First, ADAMTS1 directly compromises the integrity of the fibrous cap through its potent degradation of key extracellular matrix (ECM) components, particularly versican [20]. This excessive proteolytic activity creates a “degradable microenvironment,” undermining the overall structural stability of the plaque (the fibrous cap serves as the core barrier preventing plaque rupture, and its compromised integrity is a key trigger for plaque rupture). Second, ADAMTS1 drives pathological behavior in vascular smooth muscle cells (VSMCs). Its expression levels significantly increase in proliferating and migrating VSMCs [20]. In vivo models confirm that ADAMTS1 overexpression exacerbates intimal hyperplasia [20]. This “proliferation-promoting and migration-enhancing” effect on VSMCs is critically regulated by microRNAs: in atherosclerotic lesions, both miR-362-3p and miR-365b-3p expression is downregulated. These microRNAs have been identified as direct negative regulators of ADAMTS1—they suppress VSMC proliferation and migration by targeting and inhibiting ADAMTS1 expression [21,22].

Therefore, within the complex pathophysiological environment of atherosclerotic plaques, ADAMTS1 acts as a key mediator of plaque instability through dual pathways: on one hand, it erodes the ECM scaffold structure; on the other hand, it stimulates adverse responses in vascular smooth muscle cells (VSMCs). Ultimately, this disrupts plaque homeostasis, accelerates plaque progression, and increases the risk of rupture. This pathogenic role of ADAMTS1-mediated proteoglycan remodeling extends from spontaneous plaque rupture to the clinical setting of iatrogenic restenosis, where stent injury triggers aggrecan accumulation alongside a dynamic shift in ADAMTS protease expression from ADAMTS1/5 to ADAMTS4 [23].

### 3.2. In Heart Failure Post-MI

To understand the role of ADAMTS1 in post-myocardial infarction (post-MI) remodeling, it is essential to consider the specific context of reparative cardiac fibrosis. Following ischemic injury, the precise regulation of collagen deposition is critical, as this process forms replacement scar tissue that prevents ventricular rupture, a phenomenon termed “replacement fibrosis.” However, when this process becomes uncontrolled, it leads to excessive and structurally disorganized collagen deposition within the myocardial interstitium (i.e., interstitial fibrosis). This results in increased myocardial stiffness and impaired diastolic function, ultimately progressing to heart failure [24]. Therefore, the integrity of the cardiac extracellular matrix (ECM) is critically important following myocardial infarction (MI). In this pathological context, ADAMTS1 is not merely a passive “bystander” molecule but an active pathological mediator. Its dysregulated expression significantly promotes adverse myocardial tissue repair and progressive deterioration of cardiac function.

The mechanisms by which ADAMTS1 exerts harmful effects in the post-myocardial infarction heart primarily focus on two core aspects: first is the previously mentioned induction of excessive degradation of the extracellular matrix (ECM); second is the direct induction of a pro-fibrotic phenotype in cardiac fibroblasts. The involvement of ADAMTS1 in pathological cardiac remodeling was first supported in an aortic banding rat model. This study revealed significantly elevated expression levels of both ADAMTS1 and ADAMTS4 in myocardial tissue [25], suggesting the potential role in the characteristic extracellular matrix dysregulation observed in heart failure. Furthermore, in a mouse model of viral myocarditis progressing to dilated cardiomyopathy (a pathological process highly similar to post-myocardial infarction cardiac remodeling), therapeutic intervention with Ad-IL17RA:Fc significantly improved cardiac function and reduced mortality. Crucially, this protective effect was directly correlated with significant downregulation of ADAMTS1 in cardiac tissue, accompanied by reduced expression levels of matrix metalloproteinase-2 (MMP-2) and collagen. This finding directly establishes a causal link between “suppressing ADAMTS1 expression” and “alleviating myocardial fibrosis and improving prognosis” [26]. This causal relationship was further supported by in vitro experimental evidence. Studies demonstrated that interleukin-17A (IL-17A), a key inflammatory factor in myocarditis pathogenesis, directly induces ADAMTS1 expression in cultured cardiac fibroblasts, simultaneously promoting myocardial fibroblast proliferation and upregulating synthesis of type I and III collagen [26]. This finding unequivocally positions ADAMTS1 at the core of the “pro-inflammatory-to-pro-fibrotic” signaling axis, revealing its pivotal mediating role in inflammation-driven myocardial fibrosis.

The pathological significance of ADAMTS1 in human myocardial infarction has been validated through autopsy studies. Researchers detected ADAMTS1 and its specific substrate, versican, a multifunctional proteoglycan, and observed their distinct distribution patterns. This suggests active versican degradation (versicanolysis) mediated by ADAMTS1 during cardiac remodeling following myocardial infarction [27], further confirming ADAMTS1′s involvement in the pathological process of human myocardial infarction. Beyond the infarct zone, ADAMTS1 is also closely associated with the broader category of “stress-induced heart failure.” A recent integrated transcriptomic analysis identified ADAMTS1 as a novel transcriptional target of Kruppel-like factor 6 (KLF6) in cardiac fibroblasts. Transforming growth factor-β (TGF-β) and angiotensin II (Ang II) are known core drivers of cardiac fibrosis. This study confirms that TGF-β and Ang II can upregulate ADAMTS1 expression in a KLF6-dependent manner [28]. More importantly, in a pressure-loaded mouse model, specific knockout of *Adamts1* in myofibroblasts significantly reduced myocardial fibrosis and restored cardiac function to normal levels—establishing a direct causal relationship for ADAMTS1′s pathogenic role in pressure-loaded heart failure [27]. This study further elucidated the core mechanism: activated by key fibrotic stimulants such as TGF-β and Ang II, ADAMTS1 ultimately drives the onset and progression of heart failure by regulating “the transformation of cardiac fibroblasts into ECM-synthesizing myofibroblasts.”

Building upon this foundation, a groundbreaking study further elucidated a novel mechanotransduction pathway driven by ADAMTS1 in post-myocardial infarction cardiac fibrosis [29]. Using endothelial cell-specific ADAMTS1 overexpression and knockout models, the study confirmed that endothelial cell-derived ADAMTS1 is a key driver of cardiac dysfunction and excessive scarring following myocardial infarction. At the mechanistic level, ADAMTS1 does not directly act on cardiac fibroblasts but modulates extracellular matrix stiffness by degrading proteoglycans. This ADAMTS1-mediated alteration of the biomechanical microenvironment is specifically sensed by integrin α8 (ITGα8) on the surface of cardiac fibroblasts, triggering their pro-fibrotic activation. Notably, among multiple integrins, ITGα8 selectively responds to the mechanical signals generated by ADAMTS1. Crucial evidence demonstrates that fibroblast-specific knockout of ITGα8 sufficiently reverses the adverse cardiac remodeling and dysfunction induced by ADAMTS1 overexpression, thereby establishing a direct causal role for this endothelial–fibroblast cross-talk axis in pathological post-MI remodeling.

The pro-fibrotic function of ADAMTS1 is finely regulated by a microRNA network. Within the aldosterone-mineralocorticoid receptor (Aldo-MR) pathway, which is one of the primary drivers of cardiac remodeling following myocardial infarction, miR-181a has been demonstrated to act as a cardioprotective regulatory molecule. Its protective effects are partially mediated through direct suppression of the *Adamts1* gene: on one hand, miR-181a knockout significantly exacerbates cardiac dysfunction and remodeling in mice post-myocardial infarction, with this effect directly correlated to increased *Adamts1* expression; On the other hand, overexpression of miR-181a exerts cardioprotective effects by suppressing *Adamts1* expression [30]. The discovery of this regulatory axis indicates that ADAMTS1 is a key downstream effector molecule in the “classic pathological pathway driving heart failure,” further highlighting its central role in post-myocardial infarction heart failure.

In summary, ADAMTS1 functions as a “signal convergence node” in the pathological progression of heart failure following myocardial infarction, integrating the regulation of three key pathological signals: inflammatory mediators (e.g., IL-17A), neurohormonal pathways (e.g., the aldosterone-mineralocorticoid receptor pathway), and pro-fibrotic factors (e.g., TGF-β, Ang II). Induction of ADAMTS1 expression establishes a “matrix degradation-promoting fibrosis” pathological microenvironment. This environment not only drives adverse ventricular remodeling but also impairs cardiac function and increases the risk of cardiac rupture following myocardial infarction. Based on these findings, targeting the ADAMTS1 pathway offers a significant therapeutic approach to delay the progression from myocardial injury to heart failure, demonstrating substantial potential for clinical translation.

### 3.3. In Aortic Aneurysm/Dissection

The role of ADAMTS proteases in the pathogenesis of aortic aneurysms has been a subject of intensive investigation, particularly regarding their function in mediating abnormal vascular remodeling, which is a common feature of both thoracic aortic aneurysms (TAA) and abdominal aortic aneurysms (AAA). Previous literature has demonstrated that multiple members of the ADAMTS family are implicated in disrupting the extracellular matrix (ECM) homeostasis within the aortic wall, a central event in aneurysm formation and progression [31]. The pathological significance of this protease family is underscored by their emerging role as key regulators in TAAD, with growing interest in their potential as biomarkers and therapeutic targets [32]. This dysregulation prominently involves the impaired turnover of key proteoglycan substrates, versican and aggrecan. This review primarily focuses on ADAMTS1.

The role of ADAMTS1 in aortic aneurysms and dissections is complex and highly dependent on anatomical location and disease pathogenesis. In thoracic aortic aneurysm and dissection (TAAD), existing evidence consistently indicates that ADAMTS1 is a potent driver of disease progression. Conversely, its role in abdominal aortic aneurysm (AAA) remains unclear, suggesting that these two conditions may involve distinct pathophysiological mechanisms.

#### 3.3.1. ADAMTS1: A Driver of Thoracic Aortic Disease

The association between ADAMTS1 and thoracic aortic aneurysms was first suggested by early genomic profiling studies. As early as 2005, Taketani, T. et al. applied cDNA microarray technology to TAA specimens, revealing sustained upregulation of ADAMTS1 mRNA in human aneurysmal tissue compared to adjacent normal aorta [33]. Despite limitations in technology and sample size at the time, this pioneering study provided crucial preliminary clinical relevance. This finding has since been validated and mechanistically explored through subsequent functional studies. Extensive human research evidence consistently demonstrated a strong association between ADAMTS1 and TAAD [34,35,36]. The pathological process is now known to involve the impaired clearance and massive co-accumulation of versican and aggrecan [37].

In TAAD, the cellular sources of ADAMTS1 primarily include infiltrating inflammatory cells (particularly macrophages and neutrophils) and VSMCs [35,36], indicating that its expression is associated with both inflammatory responses and abnormal cellular synthesis functions within the aortic wall. However, the net outcome of proteolytic activity depends on the balance among multiple ADAMTS proteases. For instance, in human TAAD, despite upregulation of ADAMTS1 expression, the potent glycosaminoglycanase *ADAMTS5* is often downregulated [37]. Developmental defects in *ADAMTS5*, conversely, lead to severe aortic malformations [38]. This differential regulation suggests that despite the presence of ADAMTS1, it may not fully compensate for the absence of other critical enzymes (such as ADAMTS5) in maintaining glycosaminoglycan homeostasis. This highlights the functional non-redundancy within the ADAMTS family and the complex proteolytic imbalance observed in TAAD.

The pathological significance of ADAMTS1 is further demonstrated by its potential as a clinical biomarker. Compared to patients with acute myocardial infarction and healthy volunteers, serum ADAMTS1 levels are significantly elevated in patients with acute aortic dissection (AAD), exhibiting high diagnostic sensitivity and specificity [35,39]. Furthermore, the dynamic changes in postoperative serum ADAMTS4 levels [39] further confirm that the activity of ADAMTS family proteases is closely related to the acute phase of the disease.

Most compellingly, genetically modified mouse models provide direct causal evidence for the pathological role of ADAMTS1 in TAAD, though its specific function exhibits significant model dependency: in *Adamts1* haploinsufficient genetic models, reduced gene dosage alone suffices to induce spontaneous TAAD with pathological features resembling Marfan syndrome [14]. A key mechanism in this process involves dysregulation of the nitric oxide signaling pathway, whereby pharmacological inhibition of inducible nitric oxide synthase (NOS2) effectively prevents and even reverses aortic pathology in these mice [14]. In contrast, in a β-aminopropyonitrile (BAPN)-induced acquired TAAD model, postnatal inducible knockout of *Adamts1* significantly reduced both TAAD formation and rupture risk. This protective effect stems from ADAMTS1 deficiency suppressing early inflammatory responses, including reduced neutrophil and macrophage infiltration and decreased production of inflammatory mediators, revealing ADAMTS1′s pivotal role in driving destructive early inflammation in the aorta [40].

#### 3.3.2. ADAMTS1: A Unique Role in AAA

The role of ADAMTS1 in aortic aneurysms appears highly context-dependent, exhibiting significant differences and greater complexity in abdominal aortic aneurysms (AAA) compared to thoracic aortic aneurysms (TAAD). Although one proteomics study of human AAA-derived vascular smooth muscle cells (VSMCs) identified ADAMTS1 as “one of the most significantly up-regulated proteins” among extracellular matrix (ECM) remodeling-associated proteins [41]. However, other studies suggest uncertainty regarding its function.

mRNA analysis has shown that ADAMTS1 is one of the most significantly downregulated metalloproteinases in human AAA tissue compared to normal aortic tissue [31,42]. Interestingly, despite reduced mRNA levels, protein-level detection revealed increased ADAMTS1 expression in AAA tissue, primarily localized in regions populated by VSMCs and macrophages [42]. This discrepancy between mRNA and protein levels suggests the presence of significant post-transcriptional regulatory mechanisms within the AAA microenvironment.

Functionally, unlike its role in TAAD, transgenic overexpression of *Adamts1* in experimental AAA models did not alter aortic diameter, collagen, or elastin content, suggesting ADAMTS1 may not be a key driver in AAA pathogenesis [42].

#### 3.3.3. Compensatory Mechanisms Beyond Protease Specificity

Differences in ADAMTS1 function between TAAD and AAA may be related to compensatory interactions within the ADAMTS protease family. A study of *Adamts5* knockout mice revealed that although *Adamts5* deficiency causes impaired versican processing leading to aortic dilatation, soluble ADAMTS1 levels in these mice increase accordingly [43]. However, this upregulation of ADAMTS1 failed to compensate for the loss of ADAMTS5 function, which indicates that ADAMTS5, not ADAMTS1, is the key versican-degrading enzyme maintaining homeostasis in mouse aortas [43]. This finding highlights two core characteristics of ADAMTS family proteases: substrate specificity and functional non-redundancy. These properties may lead to differential activation of ADAMTS1 in thoracic versus abdominal aortic diseases, resulting in distinct pathological effects.

Overall, ADAMTS1 plays a clearly defined role as a key pathological mediator in TAAD, driving disease progression by degrading versican, inducing inflammatory cell infiltration, and dysregulating NO signaling pathways. Its strong association with human TAAD and demonstrated causal effects in animal models position it as a potentially important therapeutic target for TAAD. In contrast, the role of ADAMTS1 in AAA is more complex, potentially representing a secondary response to disease progression rather than an initial driver, thereby highlighting the fundamental biological differences between thoracic and abdominal aortic pathologies.

### 3.4. In Pulmonary Arterial Hypertension

Although evidence directly linking ADAMTS1 to pulmonary arterial hypertension (PAH) continues to accumulate, its known functions in extracellular matrix (ECM) remodeling and inflammatory regulation make it a highly plausible participant in PAH pathogenesis. The core features of PAH: perivascular inflammation, severe ECM dysregulation, and the resulting pulmonary arterial stiffness, all belong to pathological processes in which ADAMTS1 has been demonstrated to be deeply involved in other cardiovascular diseases.

A central pathological feature of PAH is the self-reinforcing vicious cycle formed by “endothelial dysfunction–inflammation–vascular remodeling” [44]. Within this cycle, abnormal hemodynamic forces and initial injury trigger the adhesion and infiltration of innate and adaptive immune cells into the pulmonary vascular wall. These inflammatory cells release abundant cytokines and proteases, which in turn drive the activation and proliferation of local pulmonary vascular cells [44,45]. Evidence from aortic disease research indicates that ADAMTS1 expression is strongly induced by proinflammatory cytokines (e.g., IL-17, TGF-β) and serves as a known substrate for macrophages and neutrophils [35]. This suggests that ADAMTS1 may similarly be upregulated in inflamed pulmonary vessels. Following ADAMTS1 expression, it cleaves key ECM proteoglycans such as versican, disrupting vascular matrix integrity. This creates conditions for enhanced immune cell migration and phenotypic switching of smooth muscle cells and fibroblasts, ultimately accelerating vascular remodeling.

The most significant role of ADAMTS1 in PAH lies in its promotion of pulmonary arterial stiffness, an emerging key prognostic indicator in PAH and a core driver of disease progression [44,46]. Vascular stiffness in PAH arises from multiple mechanisms, including ECM accumulation, collagen cross-linking, and vascular wall thickening [44,46,47]. As an enzyme that degrades versican (versicanase) and aggrecan (aggrecanase), ADAMTS1-mediated proteolysis serves as a crucial initiating step in ECM remodeling: the degradation of bulky proteoglycans like versican fundamentally alters the matrix structure. This degradation not only weakens the mechanical integrity of the vascular wall but also creates a permissive environment for subsequent deposition and pathological cross-linking of type I collagen. The abnormal deposition and cross-linking of type I collagen are the direct causes of irreversible vascular stiffening and increased pulmonary vascular resistance [46,47]. Thus, ADAMTS1 may serve as an “initiator” of ECM degradation, and this early degradation lays the groundwork for subsequent adverse fibrotic stiffening.

Furthermore, the dysregulated ECM microenvironment shaped by proteases like ADAMTS1 can activate pro-fibrotic and pro-proliferative signaling pathways. Among these, the stiff ECM itself acts as a potent mechanosensitive signaling activator, triggering mechanosensitive pathways such as YAP/TAZ (which has been implicated in PAH pathogenesis [46]). These pathways further cross-talk with core PAH drivers (e.g., TGF-β/BMPR2 pathway), collectively promoting pulmonary vascular smooth muscle cell survival and proliferation, as well as fibroblast activation. This ultimately leads to increased collagen synthesis and adverse vascular remodeling [45,46].

Although the role of ADAMTS1 in PAH requires definitive validation in PAH models, its established biological functions strongly suggest involvement in three core pathological processes of PAH: inflammation, ECM remodeling, and vascular stiffness. It is hypothesized that ADAMTS1 may occupy a pivotal position at the intersection of “inflammatory signaling and matrix degradation,” providing a pathological basis for progressive pulmonary arterial stiffness and increased right ventricular afterload by mediating destructive ECM remodeling. Therefore, targeting ADAMTS1 and its associated versican degradation pathway may represent a novel therapeutic strategy to break the vicious cycle of PAH and improve vascular stiffness.

## 4. Mechanisms Underlying the Dual Role

The dual protective and pathogenic functions exhibited by ADAMTS1 are context-dependent and not random phenomena, but rather the inevitable outcome of its position within a complex, multi-tiered regulatory network. The ultimate role played by ADAMTS1 depends on the precise integration of transcriptional, post-transcriptional, and cellular signaling, with its functional outcome ultimately determined by specific substrate interactions.

### 4.1. Transcriptional Regulation Orchestrating Responses to Diverse Stimuli

The expression of ADAMTS1 is highly responsive to various pathophysiological stimuli, and this sensitivity determines its initial activation under specific conditions, as summarized in Table 1. The key transcription factors and pathways regulating its expression are as follows:

#### 4.1.1. Hypoxia and the HIF-1α Pathway

In endothelial cells, ADAMTS1 is a hypoxia-specific “transient early response gene.” Hypoxia-inducible factor-1α (HIF-1α) can directly bind its promoter region to activate ADAMTS1 expression [48]. This mechanism closely links ADAMTS1 to ischemic diseases such as myocardial infarction, as local hypoxia induced by ischemia rapidly triggers ADAMTS1 expression, participating in early pathophysiological responses.

#### 4.1.2. Inflammatory Cytokines as Specific Regulators

The inflammatory microenvironment regulates ADAMTS1 expression through “cell type-specific” and “cytokine-specific” mechanisms: In macrophages, which are the characteristic cells within atherosclerotic plaques, the synergistic action of TL1A and IL-17 upregulates ADAMTS1 expression [49]; Conversely, transforming growth factor-β (TGF-β) induces its expression, while interferon-γ (IFN-γ) suppresses it, demonstrating bidirectional regulation [49]. The anti-atherosclerotic cytokine IL-33 inhibits ADAMTS1 expression in human macrophages via the ERK/JNK/PI3K signaling pathway [50]. In cardiac myofibroblasts, the proinflammatory cytokine interleukin-1α (IL-1α), a core inflammatory mediator following myocardial infarction (post-MI), paradoxically downregulates ADAMTS1 expression at both mRNA and protein levels [50]. These discrepancies fully illustrate the complexity of cytokine regulation of ADAMTS1.

#### 4.1.3. Profibrotic and Hormonal Signaling

ADAMTS1 is a core target gene in multiple pro-fibrotic signaling pathways: in cardiomyocytes, ADAMTS1 is a direct early target gene of the aldosterone-mineralocorticoid receptor (Aldo-MR) pathway [51]; In cardiac fibroblasts, the pro-fibrotic factors TGF-β and angiotensin II (Ang II) can upregulate ADAMTS1 expression via the transcription factor Kruppel-like factor 6 (KLF6), KLF6 directly binds the ADAMTS1 promoter and activates its transcription [28]. This indicates that ADAMTS1 occupies a central position in the pro-fibrotic signaling cascade, directly participating in the tissue fibrosis process.

### 4.2. Post-Transcriptional and Post-Translational Regulation

Beyond the transcriptional level, ADAMTS1 activity is finely regulated through modulation of its mRNA stability and enzyme activation processes (Table 2).

ADAMTS1 is integrated into miRNA-mediated post-transcriptional regulatory networks: miR-181a directly targets ADAMTS1, suppressing its expression to inhibit the pro-fibrotic aldosterone-mineralocorticoid receptor (Aldo-MR) pathway, thereby acting as a “cardiac protective brake” [30]; Other miRNAs (e.g., miR-362-3p, miR-365b-3p) similarly ensure ADAMTS1 expression remains within precise boundaries.

Post-translational processing further modulates ADAMTS1 activity: it is initially synthesized as an inactive proenzyme requiring furin-mediated proteolytic cleavage for activation [52]. The proteolytic activity of activated ADAMTS1 can be directly inhibited by endogenous inhibitors, with tissue inhibitor of metalloproteinase-3 (TIMP-3) being the most critical suppressor [52,53]. The “equilibrium between active ADAMTS1 and TIMP-3” in the extracellular space is the core factor determining the net proteolytic activity.

### 4.3. Cell Sources and Microenvironmental Context

The biological effects of ADAMTS1 expression are intrinsically linked to its cellular origin and surrounding microenvironment (Table 3).

The cellular origin of ADAMTS1 is a key determinant of its functional role in cardiovascular pathophysiology. ADAMTS1 secreted by different cells produces distinctly different biological effects due to variations in cellular properties and regulatory pathways. ADAMTS1 secreted by endothelial cells under hypoxic conditions [48] exhibits markedly different functions compared to ADAMTS1 secreted by macrophages in inflammatory plaques [16,49,50] or activated cardiac fibroblasts in failing hearts [28]. For instance, in aortic dissection, ADAMTS1 secreted by infiltrating macrophages and neutrophils correlates with versican degradation and disease progression [35]. Yet in Stanford type B aortic dissection patients, ADAMTS1 expression in monocytes is paradoxically reduced, suggesting its role may dynamically shift across disease stages [16].

This cell-specific paradigm is further validated in the recently identified “ADAMTS1-integrin α8” mechanotransduction axis of myocardial infarction remodeling [29]. This pathway reveals a sophisticated indirect mechanism: ADAMTS1 derived from endothelial cells does not directly transmit signals through traditional proteolytic feedback pathways, but instead exerts its effects by remodeling the biomechanical properties of the extracellular matrix. The resulting changes in matrix stiffness are specifically recognized by ITGα8 on cardiac fibroblasts and decoded as potent activation signals, driving the fibroblasts toward a pro-fibrotic phenotype. This mechanism demonstrates that the functional output of ADAMTS1 depends not only on its cellular origin but, more critically, is regulated by the receptor repertoire expressed by target cells within the microenvironment.

The net effect of ADAMTS1 activity depends on its balance with local inhibitors: Pericyte regulation exemplifies a protective mechanism. Upon sensing shear stress, pericytes upregulate TIMP-3 expression to antagonize endothelial-derived ADAMTS1, thereby maintaining vascular stability [53]. Imbalance in the ADAMTS1-TIMP3 axis leads to pathological ECM degradation, driving disease progression. Furthermore, the overall “protease-antiprotease” equilibrium within the ECM determines whether ADAMTS1-mediated remodeling constitutes “constructive repair” or “destructive injury” [52].

### 4.4. The Spatiotemporal Dynamics of ADAMTS1 Expression

The “temporal pattern” and “expression level” of ADAMTS1 are key determinants of its function:

- Transient acute upregulation: Rapid upregulation of ADAMTS1 in endothelial cells under hypoxic conditions [48], potentially exerting a protective role by moderately loosening the ECM to facilitate adaptive responses such as angiogenesis;
- Persistent chronic overexpression: Under sustained fibrotic signaling driven by factors like KLF6 [28] or Aldo-MR [30,51], ADAMTS1 exhibits prolonged high expression, leading to excessive ECM degradation and detrimental remodeling—a scenario commonly observed in post-myocardial infarction heart failure and progressive aortic aneurysms;
- Similar phenomena are observed in age-dependent differences in ischemia preconditioning: ADAMTS1 expression levels are higher and persist longer in aged rats, directly correlating with poorer prognosis [54], further confirming the functional impact of “expression duration and intensity.”

### 4.5. Substrate Specificity and Novel Non-Canonical Functions

**Figure 2:**
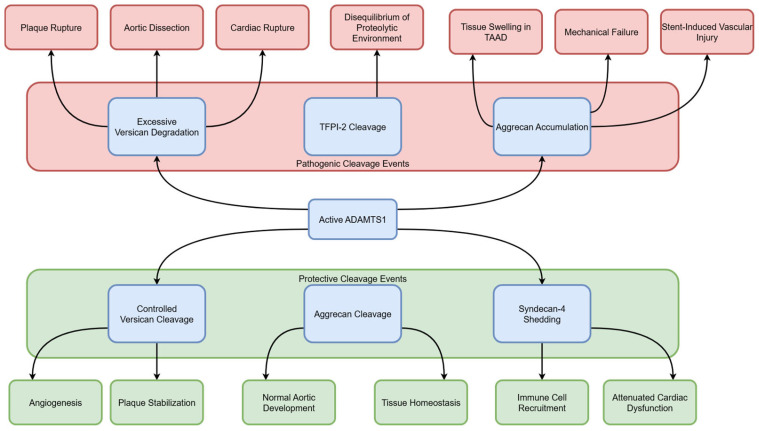
Substrate-specific functional outcomes of ADAMTS1 determine its dual roles. ADAMTS1 exerts context-dependent protective and pathological effects through its interactions with specific substrates. Its protective roles include maintaining plaque stability and inhibiting angiogenesis via versican cleavage, ensuring normal development through controlled aggrecan cleavage, and attenuating cardiac dysfunction via syndecan-4 shedding. Conversely, its pathological roles involve promoting plaque rupture, aortic dissection, and cardiac rupture through versican cleavage; contributing to tissue swelling and mechanical failure via aggrecan accumulation in TAAD; and disrupting proteolytic balance by cleaving TFPI-2. TAAD, thoracic aortic aneurysm and dissection; LPS, lipopolysaccharide.

The dual role of ADAMTS1 also stems from its diverse substrate spectrum and non-proteolytic functions (Figure 2). As a central executor of ECM remodeling, ADAMTS1 primarily targets two major proteoglycans: versican, a key structural component of the vascular wall, and aggrecan, a molecule with emerging significance in cardiovascular pathology [11]. Proteolysis, as an irreversible post-translational modification, exerts a profound impact on the function, abundance, and localization of these proteoglycans, thereby dictating their ultimate biological outcomes [55].

#### 4.5.1. The Role of Classic ECM Substrates: Versican and Aggrecan

ADAMTS1 cleavage of its primary substrates, versican and aggrecan, serves as a common core mechanism for its dual roles. The functional outcome is critically dependent on the biological context and the extent of cleavage.

- Versican: ADAMTS1 cleavage of versican serves as a common core mechanism for its involvement in both “protective” and “pathological” processes. Protective role: Potentially maintains plaque stability by inhibiting excessive angiogenesis; Pathological role: Mediates plaque rupture, aortic dissection, and cardiac rupture [20,27,36].
- Aggrecan: Similarly, ADAMTS1-mediated aggrecanolysis plays a context-dependent role. The pathological co-accumulation of aggrecan alongside versican is a hallmark of thoracic aortic aneurysm and dissection (TAAD), where it contributes to tissue swelling and mechanical failure [37]. Furthermore, in stent-induced vascular injury, aggrecan accumulation driven by a reprogrammed ADAMTS protease profile (including ADAMTS1) exemplifies its role in hyper-proliferative remodeling [23].

This duality stems from the fact that both versican and aggrecan are large, hydrophilic proteoglycans that define tissue volume and mechanical properties. Their moderate preservation is crucial for structural integrity, whereas their excessive degradation or accumulation fundamentally compromises the ECM’s load-bearing capacity. The precision of aggrecan cleavage is particularly critical, as studies using neo-epitope antibodies have demonstrated that specific cleavage fragments generated by ADAMTS proteases are essential for normal aortic wall development and homeostasis, while altered fragment profiles are linked to disease [38].

#### 4.5.2. Novel Substrates and Non-Classical Interactions

The non-ECM substrate interactions of ADAMTS1 further expand its functional complexity: (1) Interaction with TFPI-2: Tissue factor pathway inhibitor-2 (TFPI-2) has been demonstrated to be both a binding partner and substrate of ADAMTS1 [56]. By cleaving TFPI-2, ADAMTS1 indirectly alters the proteolytic environment around cells, potentially disrupting homeostatic balance in atherosclerosis and cancer; (2) Interaction with Syndecan-4: ADAMTS1 participates in the shedding of Syndecan-4 (multiligand proteoglycan-4)—a process that, while promoting immune cell recruitment, paradoxically alleviates cardiac dysfunction in lipopolysaccharide (LPS)-induced inflammation [57]. This seemingly contradictory effect further highlights the complexity of ADAMTS1′s functions.

ADAMTS1′s “dual nature” is not contradictory but rather reflects its role as a “highly regulated node” within the cardiovascular system’s adaptive response network. At the transcriptional level, it integrates hypoxia, inflammation, fibrosis, and hormonal signaling. At the expression and activity levels, it undergoes fine-tuned regulation by miRNAs and endogenous inhibitors, ultimately achieving precise control of expression and activity through “cell-specific” and “environment-specific” mechanisms. Whether ADAMTS1 ultimately exerts physiological protective or pathological damaging effects depends on the duration and magnitude of its expression, as well as the substrate types it acts upon within specific tissue microenvironments. Deep understanding of this complex regulatory network is a critical prerequisite for developing ADAMTS1-targeted therapeutic strategies while avoiding interference with its essential homeostatic functions. Furthermore, understanding the function of ADAMTS1 requires placing it within the context of the entire ADAMTS protease family. Complex compensatory relationships exist among family members, exemplified by the compensatory upregulation of ADAMTS1 observed in *Adamts5*-deficient models [43]. However, such compensation is often incomplete due to strict substrate specificity and spatiotemporal expression differences, ultimately leading to specific pathological phenotypes. Therefore, future research must approach the regulation of cardiovascular ECM remodeling from a “protease network” perspective, rather than solely focusing on individual molecules.

## 5. Therapeutic Potential and Future Perspectives

In summary, ADAMTS1 serves not only as a key mediator of cardiovascular remodeling but also as a highly promising therapeutic target. However, its dual role, which is context-dependent, presents both challenges and opportunities for precision medicine. Future research should shift from “broad-spectrum inhibition” to “fine-tuned regulation” based on a deeper understanding of its mechanisms, enabling precise intervention in ADAMTS1 function.

### 5.1. ADAMTS1: A Biomarker for Risk Stratification

The stable association of ADAMTS1 with specific cardiovascular pathologies highlights its potential as a biomarker. From a genetic perspective, the rs402007 polymorphism and Ala227Pro variant of ADAMTS1 identify individuals at significantly elevated risk for coronary events, who thus typically derive greater benefit from statin therapy [58,59,60]. This pharmacogenetic interaction suggests that ADAMTS1 genotyping can guide primary prevention strategies, enabling personalized statin prescribing to maximize therapeutic efficacy. In acute disease settings, serum ADAMTS1 levels demonstrate high diagnostic accuracy for type A acute aortic dissection (AAD), with diagnostic performance comparable to established biomarkers like D-dimer [39]. Furthermore, ADAMTS1 is included in the “11-gene immune-related signature” for acute myocardial infarction (AMI) diagnosis, further demonstrating its value in multi-biomarker panels [61]. More importantly, the potential of ADAMTS1 as a dynamic biomarker has also been validated across disease domains. For instance, in patients with primary hyperparathyroidism complicated by cardiovascular stress, the significant reduction in serum ADAMTS1 levels following parathyroidectomy suggests its utility in quantitatively assessing the degree of cardiovascular stress relief following surgical intervention [62]. However, establishing ADAMTS1 as a universal circulating biomarker remains challenging. A recent study in patients undergoing elective coronary angiography indicated that plasma ADAMTS1 levels did not demonstrate significant diagnostic or grading value for assessing the presence and severity of coronary artery disease, unlike interleukin-8 (IL-8) [63]. This finding suggests that the utility of ADAMTS1 as a soluble biomarker may be disease-specific, with its release kinetics during acute vascular events (e.g., AAD, AMI) potentially differing fundamentally from those observed in chronic, stable atherosclerosis (e.g., CAD).

### 5.2. Therapeutic Targeting of ADAMTS1: Prospects and Challenges

The core challenge in targeting ADAMTS1 therapeutically lies in its dual functionality: adopting a global inhibition strategy (as exemplified by the protective effect of “lauric acid-mediated macrophage ADAMTS1 downregulation” proposed in reference [64]) may inadvertently disrupt its critical homeostatic functions (such as maintaining vascular integrity via the ADAMTS1-NOS2 axis [14]). This dilemma parallels historical failures in developing broad-spectrum matrix metalloproteinase (MMP) inhibitors [65]. Current research focuses on more refined indirect intervention strategies, primarily categorized into three approaches.

#### 5.2.1. Targeting Upstream Regulators

Interventions targeting upstream regulatory pathways have demonstrated potential. For instance, in *Adamts1*-deficient mice and mouse models of Marfan syndrome, targeting the NOS2 pathway reverses aortic pathology—this strategy avoids disrupting ADAMTS1′s homeostatic function by “bypassing ADAMTS1 to intervene directly” [14]. Furthermore, the discovery that the ADAMTS protease profile is dynamically reprogrammed (e.g., shifting from ADAMTS1/5 to ADAMTS4) in response to vascular injury such as stenting [23], provides a compelling rationale for targeting these upstream regulatory networks rather than the protease itself. This approach could allow for the correction of the overall proteolytic imbalance without completely inhibiting specific ADAMTS members.

#### 5.2.2. Drug Repurposing: Existing Drugs Modulating ADAMTS1

The application of statins offers a novel approach for ADAMTS1 targeting: in susceptible subgroups carrying specific ADAMTS1 genotypes, statins may exert partial therapeutic benefits by modulating ADAMTS1-related pathways [58,59,60]. This suggests that drug repurposing strategies could be employed to explore the regulatory potential of existing drugs on ADAMTS1, thereby reducing the costs and risks associated with developing new drugs.

#### 5.2.3. Precision Targeting with Engineered Inhibitors

Drawing on research strategies for other metalloproteinases [65], the development of engineered protein inhibitors targeting specific exosites of ADAMTS1 holds promise for achieving “substrate-specific inhibition”, which refers to selectively suppressing only the pathological cleavage functions of ADAMTS1 (such as the excessive degradation of versican, which destabilizes plaques) while preserving its protective cleavage activities (such as maintaining vascular homeostasis).

### 5.3. Future Research Directions

To overcome existing limitations and fully unlock the therapeutic potential of ADAMTS1, future research should focus on the following specific and actionable directions:

#### 5.3.1. Elucidating Cell-Type-Specific Mechanisms

There is an urgent need to establish cell-specific knockout models within this field (such as ADAMTS1 knockout in macrophages, endothelial cells, vascular smooth muscle cells, and fibroblasts) to clarify the unique roles of ADAMTS1 from different cellular origins in disease pathogenesis and progression. Recent phosphoproteomics studies have identified ADAMTS1 as a differentially expressed protein in vascular smooth muscle cells derived from abdominal aortic aneurysms (AAA) [41], laying the groundwork for subsequent investigations. However, functional validation remains necessary to clarify its specific role in AAA pathogenesis.

#### 5.3.2. Defining the ADAMTS1 Substrate and Functional Landscape

Beyond the known substrate versican, the complete substrate profile of ADAMTS1 in the cardiovascular system remains unclear. Novel substrates require identification through systematic approaches such as degradomics in specific pathological scenarios, including plaque rupture and aortic dissection. For instance, the interaction between ADAMTS1 and TFPI-2 suggests it may exert effects through indirect regulation of the proteolytic microenvironment [56], a mechanism warranting further investigation. Additionally, the role of ADAMTS1 in immune responses associated with non-alcoholic fatty liver disease (NAFLD) and atherosclerosis [56], along with its regulation by non-coding RNAs [66,67], represents highly promising research directions.

#### 5.3.3. Developing Context-Specific Modulators

Based on the structural characteristics of the highly variable loop and external sites of ADAMTS1 [10], developing “substrate-selective inhibitors” or “functional blockers” is the core objective. High-throughput screening is required to identify small-molecule compounds or design biologics that inhibit pathological ADAMTS1 functions (e.g., excessive versican degradation in plaque vulnerability [4,68]) while preserving its protective roles (e.g., maintaining vascular homeostasis [14]). This represents a critical frontier for achieving precise ADAMTS1 intervention.

#### 5.3.4. Synergistic Targeting for Combination Therapy

Given the complexity of the plaque microenvironment [4] and AAA pathogenesis [31,69], ADAMTS1 is unlikely to serve as a single therapeutic target. Future research should explore synergistic targeting strategies involving other key pathways, such as co-regulating cytokines like Oncostatin-M [68] or type VIII collagen remodeling [69], to achieve more effective and stable improvements in diseased tissue states.

The research journey of ADAMTS1 from a “novel gene” to a “potential therapeutic target” reflects the evolving trends in cardiovascular research toward “mechanism refinement” and “therapeutic individualization.” Future efforts in three key areas hold promise for translating ADAMTS1 regulation into cardiovascular disease therapy: First, utilizing genetic information for patient stratification to optimize treatment regimens; Second, deepening the understanding of its cell-specific and environment-specific biological functions to identify intervention windows; Third, developing precise targeting methods to achieve fine-tuned regulation of its functions. Ultimately, these strategies may leverage ADAMTS1 regulation to provide novel therapeutic approaches for various cardiovascular diseases.

## 6. Conclusions

In summary, ADAMTS1 is a classic “double-edged molecule” in cardiovascular disease, with its ultimate function, whether protective or pathological, highly dependent on the environment. This duality is precisely regulated by the interplay of cellular origin, temporal expression dynamics, local microenvironment, and equilibrium with endogenous inhibitors. On one hand, ADAMTS1 exerts protective effects by maintaining vascular integrity, regulating inflammatory responses, and promoting early adaptive remodeling. On the other hand, dysregulated ADAMTS1 expression or activity drives disease progression through excessive degradation of the extracellular matrix (ECM), the exacerbation of adverse inflammatory responses, and the disruption of vascular homeostasis, thereby contributing to the pathophysiology of atherosclerosis, aortic aneurysms, and heart failure.

Ultimately, ADAMTS1 serves as a pivotal signaling node within the ECM homeostasis regulatory network. It integrates diverse biochemical and biomechanical signals, translating them into precise modifications of the ECM, a property that underscores ADAMTS1′s central role in tissue maintenance under physiological conditions and cardiovascular remodeling during pathological states. Therefore, future therapeutic strategies targeting this pivotal protease should not aim for broad-spectrum inhibition but rather focus on precise modulation to restore the delicate homeostatic equilibrium of ADAMTS1 within the body.

## Figures and Tables

**Table 1:** Transcriptional Regulation of ADAMTS1.

Category	Stimulus/Factor	Primary Pathophysiological Context	Effect on ADAMTS1 Expression	Core Transcription Factor/Pathway	References
Hypoxic Signal	Hypoxia	Ischemic Diseases (e.g., Myocardial Infarction)	Induction	HIF-1α	[48]
Inflammatory Cytokines	IL-17 + TL1A (synergistic)	Atherosclerotic Inflammatory Milieu		N/S	[49]
	TGF-β	Inflammatory & Profibrotic Milieu		N/S	
	IFN-γ	Inflammatory Milieu	Inhibition	N/S	
	IL-33	Atherosclerotic Milieu		ERK/JNK/PI3K	[50]
	IL-1α	Post-Myocardial Infarction Inflammation		N/S	
Pro-fibrotic & Hormonal Signaling	Aldosterone	Cardiac Pressure Overload /Fibrosis	Induction	Mineralocorticoid Receptor (MR)	[51]
	Angiotensin II (Ang II)	Cardiac Fibrosis		KLF6	[28]

**Table 2:** Fine-Tuning of ADAMTS1 at the Molecular Level.

Regulatory Level	Regulatory Factor	Mechanism of Action	Effect on ADAMTS1 Activity/Expression	References
Post-Transcriptional Regulation	miR-181a	Directly targets ADAMTS1 mRNA, acting as a “cardiac protective brake”	Suppression of expression and the pro-fibrotic Aldo-MR pathway	[30]
	Other miRNAs(e.g., miR-362-3p, miR-365b-3p)	Fine-tuning of ADAMTS1 mRNA stability	Confines expression within precise boundaries	[21,22]
Post-Translational Activation	Furin Protease	Cleaves and activates the inactive ADAMTS1 proenzyme	Activation of proteolytic function	[52,53]
Endogenous Inhibition	Tissue Inhibitor of Metalloproteinase-3 (TIMP-3)	Directly inhibits active ADAMTS1 protease; upregulated by pericytes under shear stress	Suppression of net proteolytic activity; maintains vascular stability	[52]

**Table 3:** Cellular Sources, Functional Outputs, and Pathophysiological Significance of ADAMTS1.

Cellular Source	Core Pathophysiological Context	Primary Functional Role of ADAMTS1	Resulting Pathophysiological Outcome	References
Endothelial Cells	Ischemia/Hypoxia (e.g., Myocardial Infarction)	Early Responder, Adaptive Remodeling Modulator	Facilitates potential angiogenesis via moderate ECM remodeling (Protective); its acute role is under investigation.	[48]
Macrophages	Atherosclerotic Plaques (Inflammation)	Inflammatory and Matrix-Degrading Effector	Associated with versican degradation within plaques, potentially influencing plaque stability (Dual Role).	[49]
	Aortic Dissection	Promoter of Tissue Destruction	Mediates versican degradation, directly driving disease progression.	[35]
Cardiac Fibroblasts	Cardiac Pressure Overload, Fibrosis	Executor of Pro-fibrotic Signaling	Directly participates in pathological cardiac fibrosis, contributing to heart failure.	[28]
Neutrophils/Monocytes	Aortic Dissection (Acute Phase)	Stage-Dependent Modulator	Expression levels dynamically shift across disease stages, suggesting a context-dependent functional switch.	[16]
Endothelial Cells (via non-canonical mechanism)	Post-Myocardial Infarction Remodeling	Modulator of the Mechanical Environment	Indirectly drives cardiac fibroblast activation and fibrosis via the ADAMTS1-ITGα8 mechanotransduction axis.	[29]

## Data Availability

No new data were created or analyzed in this study.
